# Identification of DNA methylation-driven genes in esophageal squamous cell carcinoma: a study based on The Cancer Genome Atlas

**DOI:** 10.1186/s12935-019-0770-9

**Published:** 2019-03-06

**Authors:** Tong Lu, Di Chen, Yuanyong Wang, Xiao Sun, Shicheng Li, Shuncheng Miao, Yang Wo, Yanting Dong, Xiaoliang Leng, Wenxing Du, Wenjie Jiao

**Affiliations:** 1 Department of Thoracic Surgery, Affiliated Hospital of Qingdao University, No. 16 Jiangsu Road, Shinan District, Qingdao, 266003 China; 2 Department of Gastroenterology, Affiliated Hospital of Qingdao University, No 16 Jiangsu Road, Shinan District, Qingdao, 266003 China

**Keywords:** Esophageal squamous cell carcinoma, Methylation, Biomarker, TCGA

## Abstract

**Background:**

Aberrant DNA methylations are significantly associated with esophageal squamous cell carcinoma (ESCC). In this study, we aimed to investigate the DNA methylation-driven genes in ESCC by integrative bioinformatics analysis.

**Methods:**

Data of DNA methylation and transcriptome profiling were downloaded from TCGA database. DNA methylation-driven genes were obtained by methylmix R package. David database and ConsensusPathDB were used to perform gene ontology (GO) analysis and pathway analysis, respectively. Survival R package was used to analyze overall survival analysis of methylation-driven genes.

**Results:**

Totally 26 DNA methylation-driven genes were identified by the methylmix, which were enriched in molecular function of DNA binding and transcription factor activity. Then, ABCD1, SLC5A10, SPIN3, ZNF69, and ZNF608 were recognized as significant independent prognostic biomarkers from 26 methylation-driven genes. Additionally, a further integrative survival analysis, which combined methylation and gene expression data, was identified that ABCD1, CCDC8, FBXO17 were significantly associated with patients’ survival. Also, multiple aberrant methylation sites were found to be correlated with gene expression.

**Conclusion:**

In summary, we studied the DNA methylation-driven genes in ESCC by bioinformatics analysis, offering better understand of molecular mechanisms of ESCC and providing potential biomarkers precision treatment and prognosis detection.

**Electronic supplementary material:**

The online version of this article (10.1186/s12935-019-0770-9) contains supplementary material, which is available to authorized users.

## Background

Esophageal carcinoma (EC) is one of the most common malignant tumors in the digestive system. It occurs mostly in the esophageal epithelium, and there are no typical clinical symptoms in the early stage of the patient. Therefore, more than 80% of EC patients have progressed to the advanced stage when they are diagnosed, which affects the prognosis of patients [1]. Esophageal cancer has two major histological subtypes, esophageal adenocarcinoma (EAC) and esophageal squamous cell carcinoma (ESCC). Among them, ESCC is the predominant subtype and accounts for 80% of all patients [2]. For the moment, the mechanism of ESCC is still not fully characterized, and the early symptoms of patients are atypical, which brings great difficulties for clinical diagnosis and therapy [3]. Similar to other malignancies, the progression of ESCC is also a complex process involving multiple factors and multiple gene mutations. Studies have shown that the changes in the molecular level of ESCC tissues are earlier than the clinical features. Therefore, early diagnosis and intervention are significant for reducing the incidence of ESCC [4].

Epigenetic changes are identified as significant contributors to cancer progression [5]. The abnormal DNF methylation is one of the most important and common epigenetic modifications, and plays key roles in regulating genome function [6]. Selective hypermethylation or hypomethylation of genes to regulate the expression of genes and form specific tissue types during development are considered to be a hallmark in developing many carcinomas [7]. In recent years, studies on methylation and tumors have gradually drawn more attention. For instance, Roy et al. analyzed the lymph node metastasis in esophageal squamous cell carcinoma and built a comprehensive methylation signature for predicting the prognosis of patients [8]. Genes including NNK, MSH3 and P16, which were stated to be methylated, associated with tumors progression [9–11]. Identification of abnormal methylated genes can explore the redundancy and instability of the esophageal carcinoma genome and provide the basis for risk prediction and targeted therapy.

The wide DNA methylation arrays and advent of deep RNA-Seq approach has significantly contributed to study the interaction between methylation and gene expression during tissue carcinogenesis and development. An integrative analysis of mRNA expression and DNA methylation studied by Kim et al. stated out the function of epigenetic changes on malignant mesothelioma cell [12]. Furthermore, in order to identify the mechanism contributed to oncogenesis, Olivier Gevaert et al. developed a novel computational algorithm called Methylmix to study abnormal methylated genes and predict transcription [13]. As a well-known cancer genome database, The Cancer Genome Atlas (TCGA) [14] provides a great genomic data with patients information, which can translate molecular information into potential clinical information. In this study, ESCC-related expressed and abnormally methylated genes were recognized based on TCGA database, and the related differential genes and expression of abnormally methylated genes in ESCC patients were clarified. We analyzed RNA-Seq transcriptomes and DNA methylation data of ESCC samples from 99 cases in TCGA. Five candidate genes (ABCD1, SLC5A10, SPIN3, ZNF69, ZNF608) were identified from 26 driven genes (p < 0.05), which could be served as independent prognostic biomarker. Additionally, ABCD1, CCDC8, FBXO17 were identified to be meaningfully correlated with prognosis by further integrative survival analysis. Besides, we found the significant correlation between methylated sites with gene expression.

## Methods

### Data acquisition and preprocessing

In this study, all data were obtained from TCGA data portal accessed on 20181108 (https://portal.gdc.cancer.gov/). Of them, the DNA methylation data was using the Illumina Infinium HumanMethylation450 platform, and beta values, ranged from 0 to 1, was quantified to indicate the levels of DNA methylation. The DNA methylation data included 3 normal samples, 96 ESCC samples. And we used transcriptome profiling data without isoform expression and miRNA expression quantification, for analyzing the gene expression of ESCC. Then, R software and packages were utilized to analyze and normalize the downloaded data to obtain differentially expressed genes (DEGs) and differentially methylated genes (DMGs). Furthermore, a total of 96 ESCC suffers had recorded clinical data and were used in further survival analysis (Additional file 1: Table S1). The data from TCGA is open-ended and publicly available.

### Integrative analysis

The DEGs and DMGs were integrated for performing an analysis via the R package MethylMix [15]. MethylMix is a program used for automatically analyzing the correlation between methylation events and gene expression [13]. Three datasets are required as input for analysis: normal DNA methylation data, cancer DNA methylation data and matched gene expression data. Then, the Methylmix identify cancer specific hyper and hypo methylated genes, which named transcriptionally predictive genes, and compute the correlation between methylated genes and related genes. A Wilcoxon rank sum test was adopted in this algorithm. And the final output of MethylMix is genes that are both transcriptionally predictive and differentially methylated states. Additionally, the differential methylation (DM) value where a negative DM value signifies hypomethylation and a positive DM value signifies hypermethylation can be used in subsequent analysis.

### Methylation-driven genes functional enrichment and pathway analysis

Gene ontology (GO) analysis was conducted on identified methylation-driven genes with methylation/expression using the DAVID database. DAVID provides integrative and systematic annotation tools for unraveling biological meaning of genes. Gene ontology (GO) analysis includes the molecular function, biological process and cellular component [16]. And we used Goplot to visualize the result.

Pathway analysis was conducted for the methylation-driven genes with ConsensusPathDB [17], which is a functional molecular interaction database, integrating information on genetic interacting signaling, protein interacting, drug-target interactions, metabolism and gene regulation in humans. Over-representation analysis was based on neighbourhood entity sets or biochemical pathways, and the pathway analysis was performed on the basis of imputed gene list. Lists of hypomethylated genes and hypermethylated genes were analyzed together. We used p value cutoff of 0.05 and minimum overlap as default settings.

### Survival analysis

Kaplan–Meier curves were used to identify the relationship between methylation-driven genes and the survival in ESCC. The independent prognostic possibility of methylation-driven genes was screened via the survival R package. The p value was obtained using the long-rank test and p < 0.05 were considered statistically significant.

To further investigate the key genes from methylation-driven genes, we combined abnormal methylation genes with the corresponding gene expression data, and the joint survival analysis was performed via the survival R package. In addition, since the key genes were obtained from the above, we merged relevant sites of methylation and corresponding gene expression data, for identifying the correlation between gene expression and key gene methylation sites.

## Results

### Identification of methylation-driven genes in ESCC

**Fig. 1:**
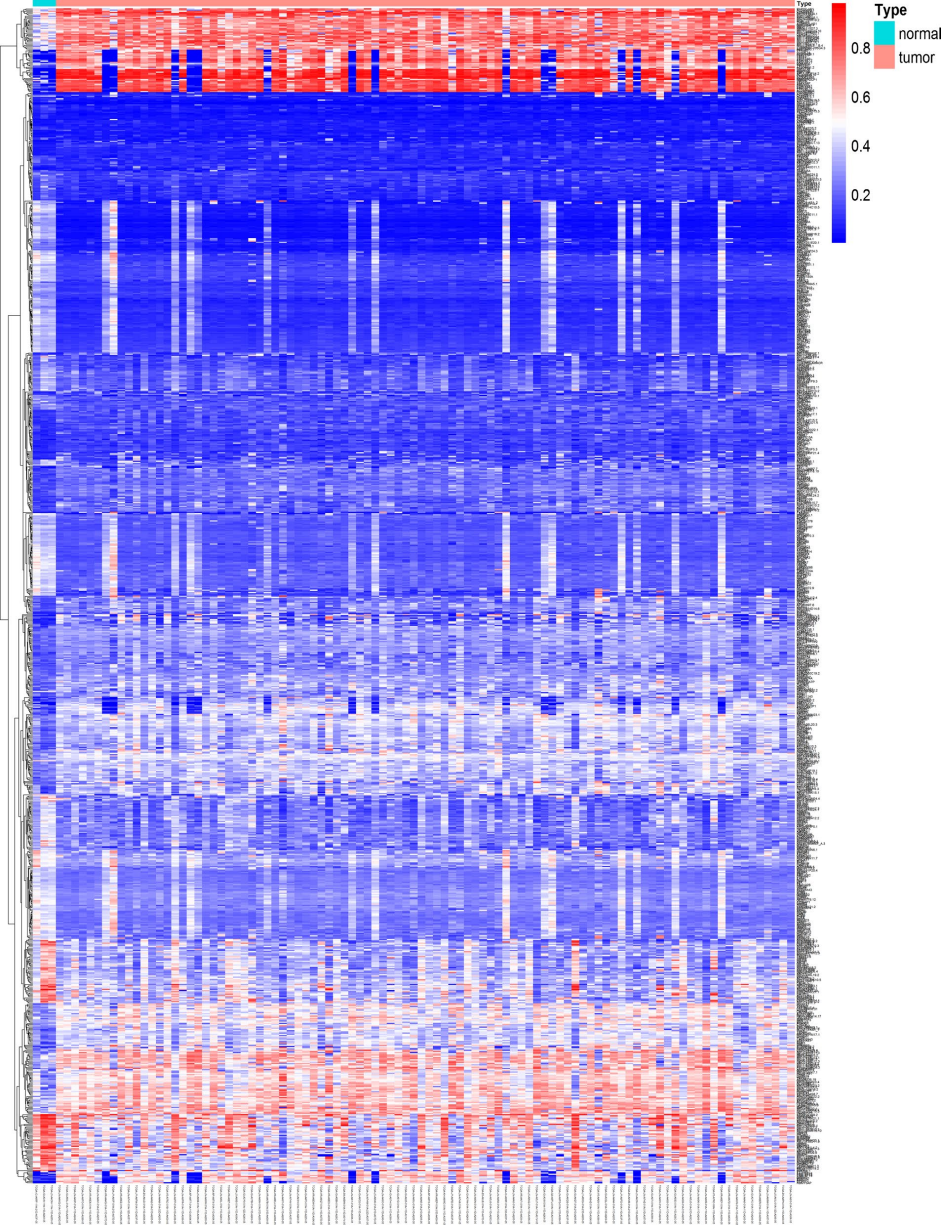
Thermal map of ESCC-related abnormal methylation genes. The color from bule to red illustrates a trend from low methylation to high methylation. *ESCC* esophageal squamous cell carcinoma

**Fig. 2:**
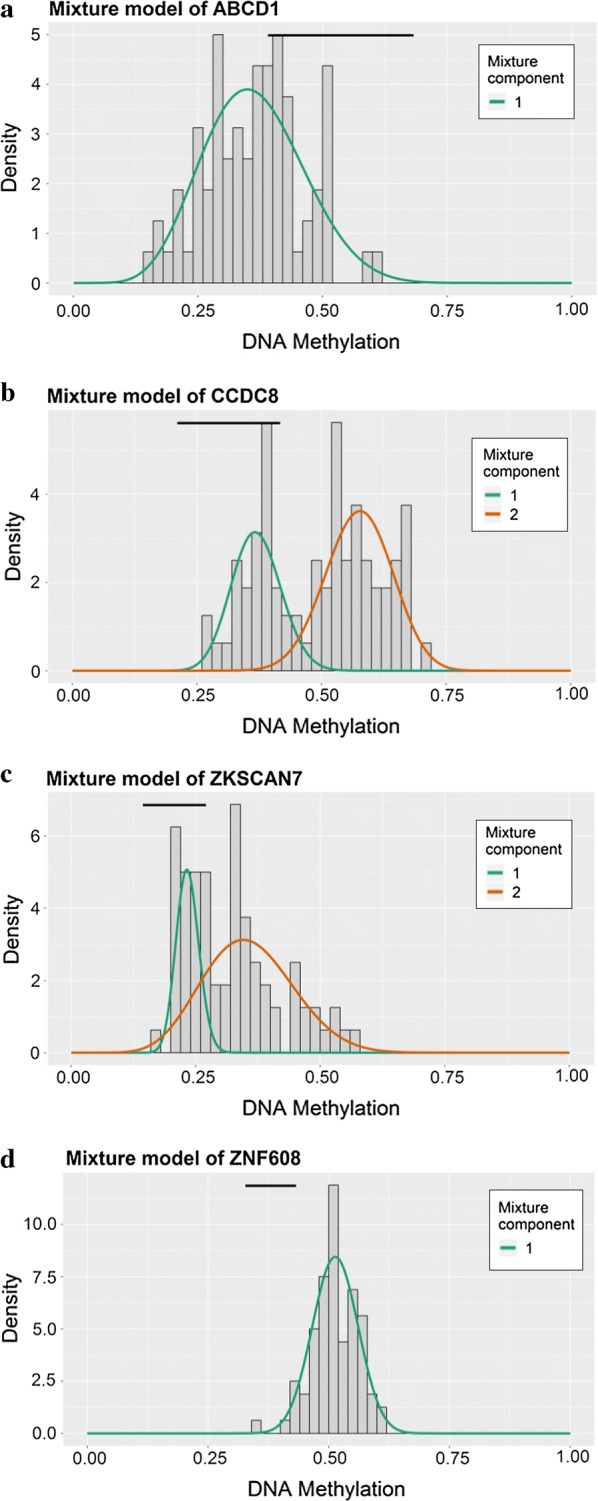
Methylmix model of DNA methylation-driven genes. The distribution maps show the methylation states of methylated genes. The histogram represents the distribution of methylation in tumor samples. The horizontal black bar demonstrates the distribution of methylation in the normal samples. The distribution of methylation degree can be clearly seen from the figure (**a**–**d**)

**Fig. 3:**
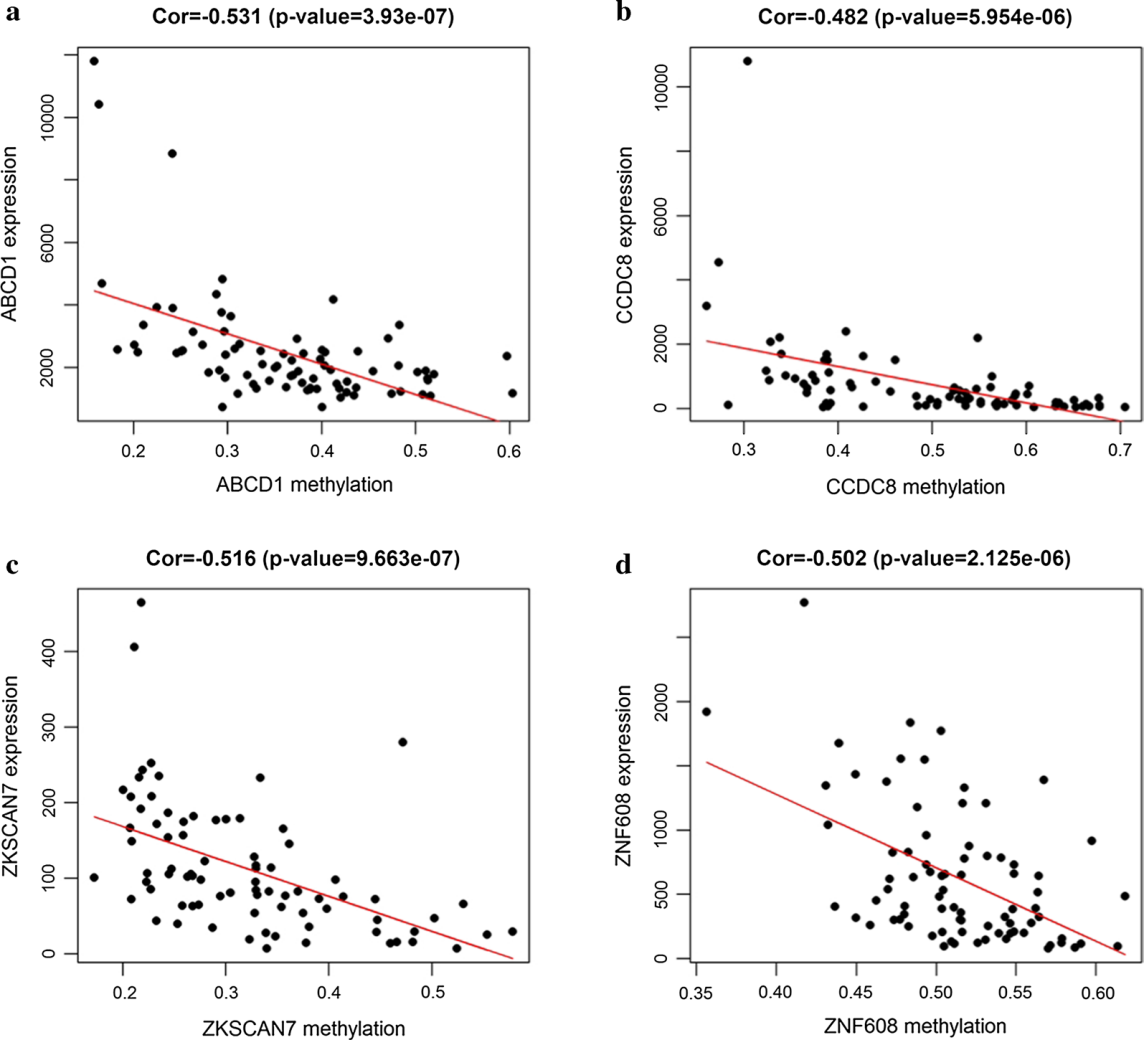
Correlation between DNA methylation and gene expression (**a**–**d**)

To study methylation-driven genes, a total of 3 normal samples and 96 sample of methylated from TCGA were included in our study. First, we used LIMMA software package for DMGs filtration (p < 0.05, |logFC ≥ 1|, and hypermethylation of 447 genes and hypomethylation of 520 genes were identified (Fig. 1). Second, edgR R package was used for identifying the DEGs in ESCC, and DEGs and DMGs were merged. Third, according to the Methylmix R package, we recombined the DEGs and DMGs and divided them into methylated cancer set, methylated normal set and gene cancer set. P < 0.05 and cor < − 0.3 were adopted for screening methylation-driven genes. Last, 26 genes were screened and we used R software to visualized the mixture model and the correlation between genes expression and degree of methylation (Table 1). Among them, 4 genes were shown in Figs. 2 and 3, while the rest were shown in Additional file 2: Figure S1 and Additional file 3: Figure S2. Furthermore, there were no significant differences between these four genes (p > 0.05).

**Table 1:** DNA methylation-driven genes in ESCC

Gene	Normal mean	Tumor mean	logFC	p value	cor
ZNF608	0.380014	0.513389	0.433998	0.004147	− 0.50173
ZNF69	0.083813	0.247231	1.560619	0.004834	− 0.50458
RGN	0.495263	0.728198	0.556136	0.005214	− 0.38076
KCNS1	0.30212	0.424639	0.491118	0.005214	− 0.45073
HNF1A	0.28917	0.418797	0.534334	0.005621	− 0.37047
DNALI1	0.245787	0.362701	0.561374	0.005621	− 0.49921
PHYHD1	0.492102	0.614435	0.320303	0.006056	− 0.56
ZNF790	0.065159	0.200691	1.622936	0.006056	− 0.40059
MCHR1	0.353121	0.48139	0.447046	0.006056	− 0.41517
G0S2	0.171841	0.35132	1.031706	0.007019	− 0.39798
SLC5A10	0.582992	0.682592	0.227548	0.007019	− 0.51545
AKR1B10	0.556426	0.463528	− 0.26353	0.007551	− 0.65002
ABCD1	0.536443	0.362675	− 0.56475	0.009368	− 0.53146
HCAR1	0.364581	0.614652	0.753531	0.011565	− 0.67757
CCDC8	0.31382	0.494431	0.655831	0.011565	− 0.4821
CD302	0.219885	0.267934	0.285128	0.012393	− 0.36856
MAEL	0.802284	0.705181	− 0.18612	0.012393	− 0.37241
DAPP1	0.484881	0.36362	− 0.4152	0.015201	− 0.40053
ZKSCAN7	0.207497	0.321143	0.630125	0.016254	− 0.51596
FBXO17	0.084149	0.138557	0.719469	0.01737	− 0.49376
SELENBP1	0.334825	0.459392	0.456318	0.017371	− 0.39778
SPIN3	0.461477	0.282616	− 0.70742	0.021134	− 0.38108
ZFP36L2	0.052163	0.158787	1.605994	0.022539	− 0.37007
IFITM2	0.150144	0.215658	0.522395	0.027245	− 0.45481
AHR	0.607399	0.752003	0.308094	0.027246	− 0.41065
ELF5	0.48324	0.597947	0.307279	0.044136	− 0.50424

### Functional enrichment and pathway analysis of methylation-driven genes

**Fig. 4:**
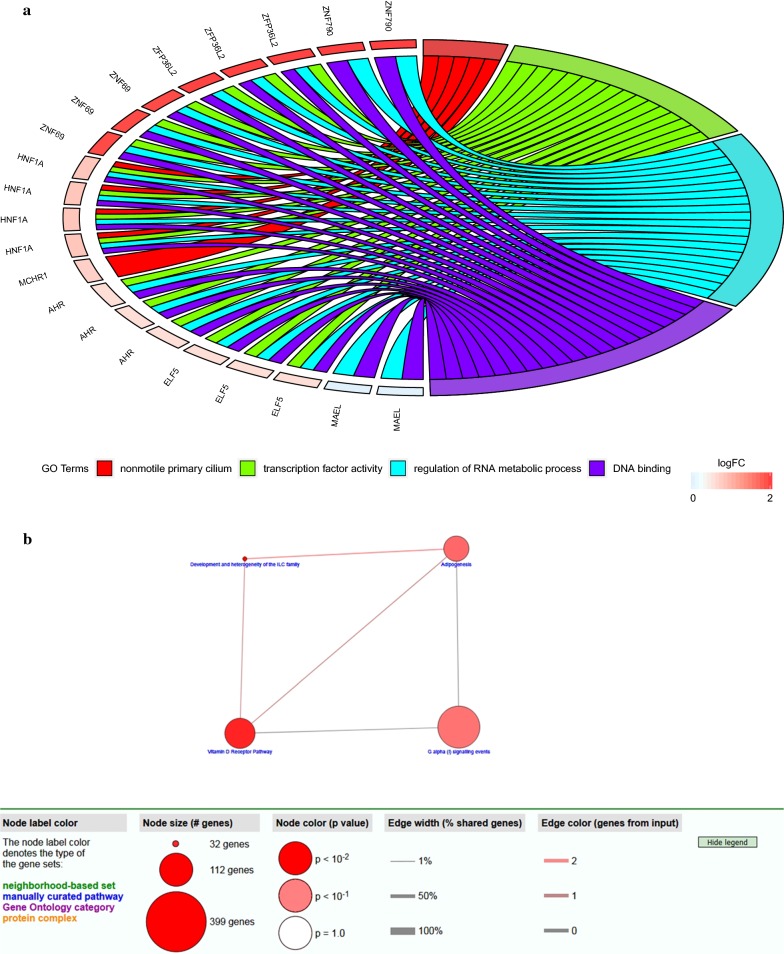
Methylation-driven genes functional enrichment and pathway analysis. **a** Gene ontology analysis of methylation-driven genes of ESCC. **b** Pathway analysis of methylation-driven genes by using ConsensuspathDB

To further investigate the function of methylation-driven genes in ESCC, we used GO enrichment analysis in DAVID. Methylation-driven genes were enriched in molecular function (MF) of DNA binding and transcription factor activity. As for cell component (CC), these genes showed enrichment in nonmotile primary cilium. Besides, biological process (BP) indicated enrichment predominantly at regulation of RNA metabolic process (Fig. 4a).

Pathway enrichment analysis revealed that methylation-driven genes were significantly linked to vitamin D receptor pathway, development and heterogeneity of the ILC family, adipogenesis and G alpha (i) signaling events (Fig. 4b).

### Prognostic assessment of methylation-driven genes in ESCC

**Fig. 5:**
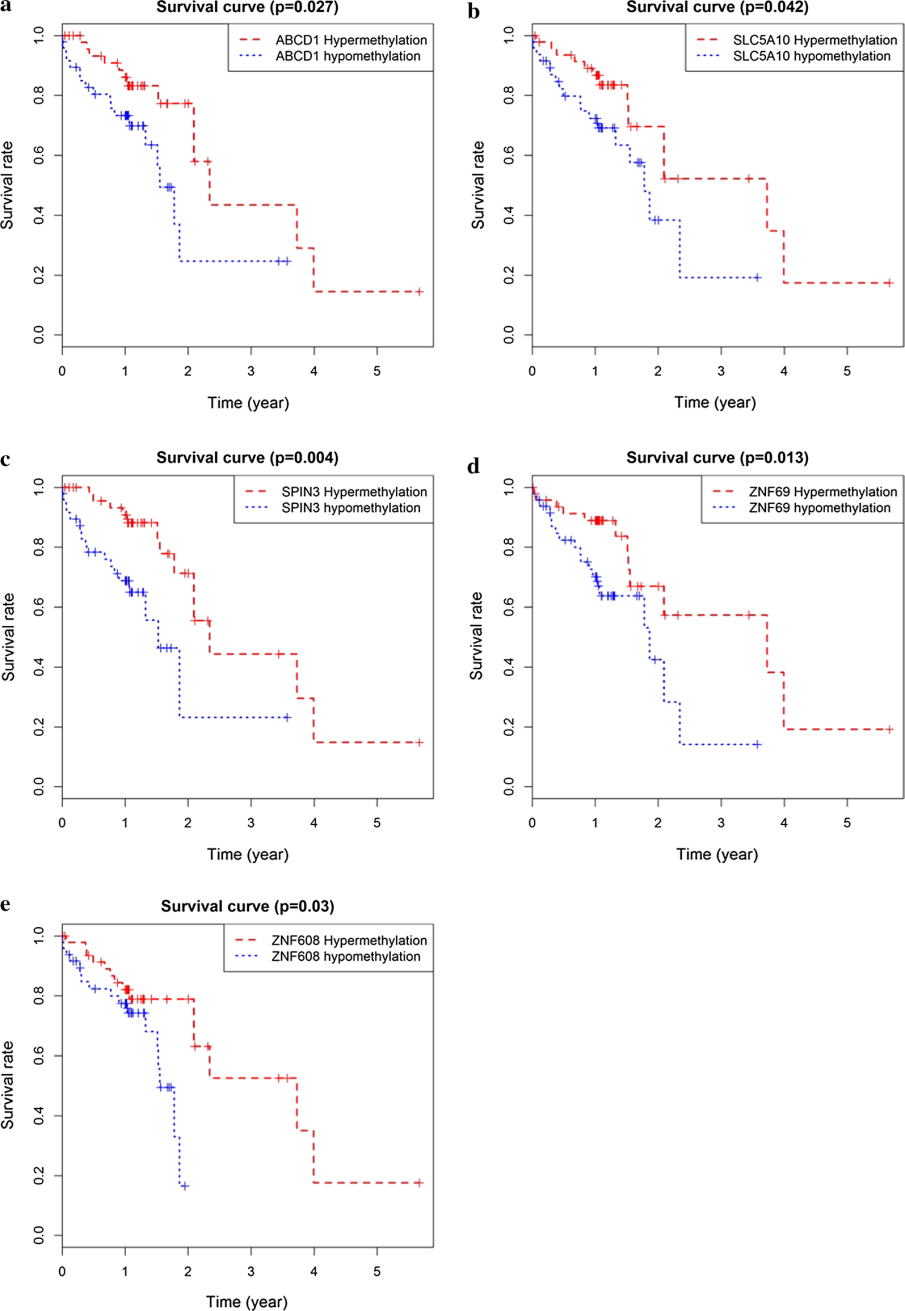
Kaplan–Meier survival curves of five independent prognostic factors (**a**–**e**)

**Fig. 6:**
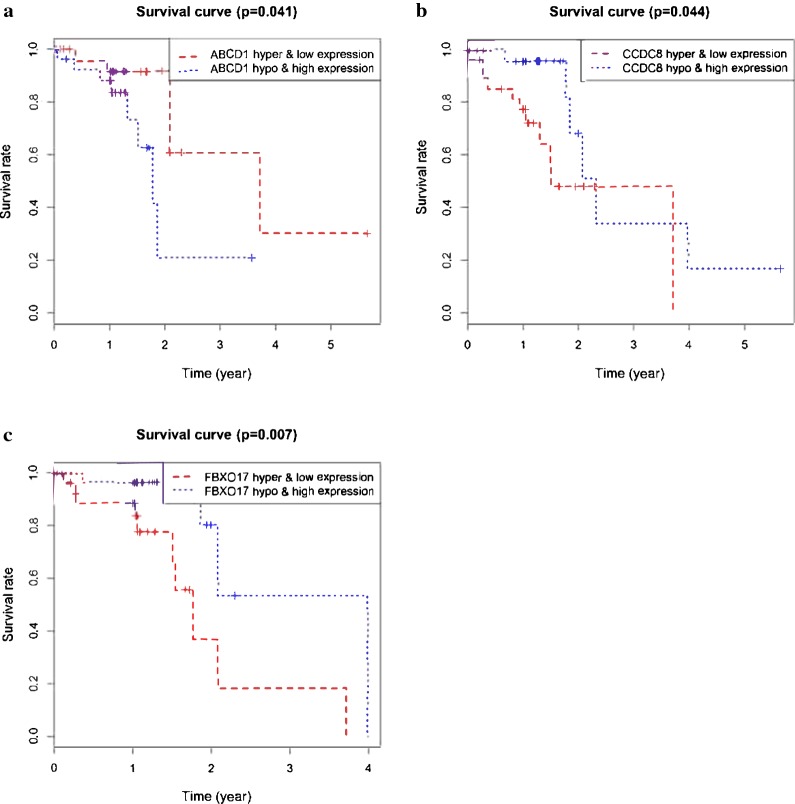
Kaplan–Meier survival curves for the integrative analysis of ABCD1, CCDC8 and FBXO17 (**a**–**c**)

**Fig. 7:**
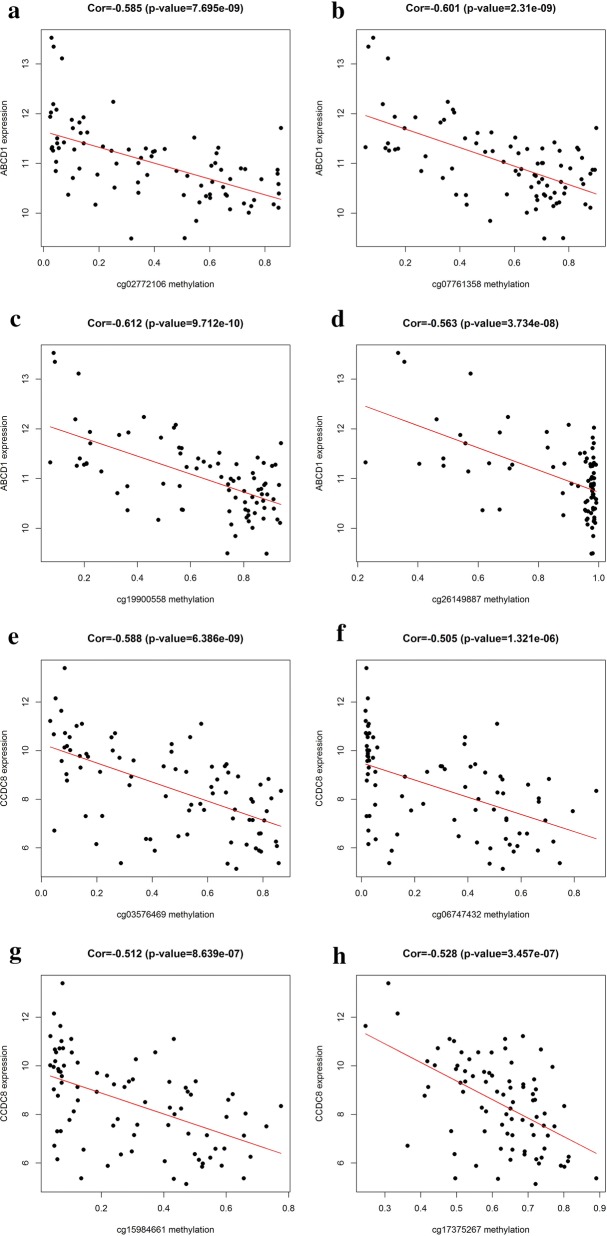
The correlation between methylated sites and gene expression. ABCD1 methylated sites with matched gene expression **a**–**d**. CCDC8 methylated sites with matched gene expression **e**–**h**

**Fig. 8:**
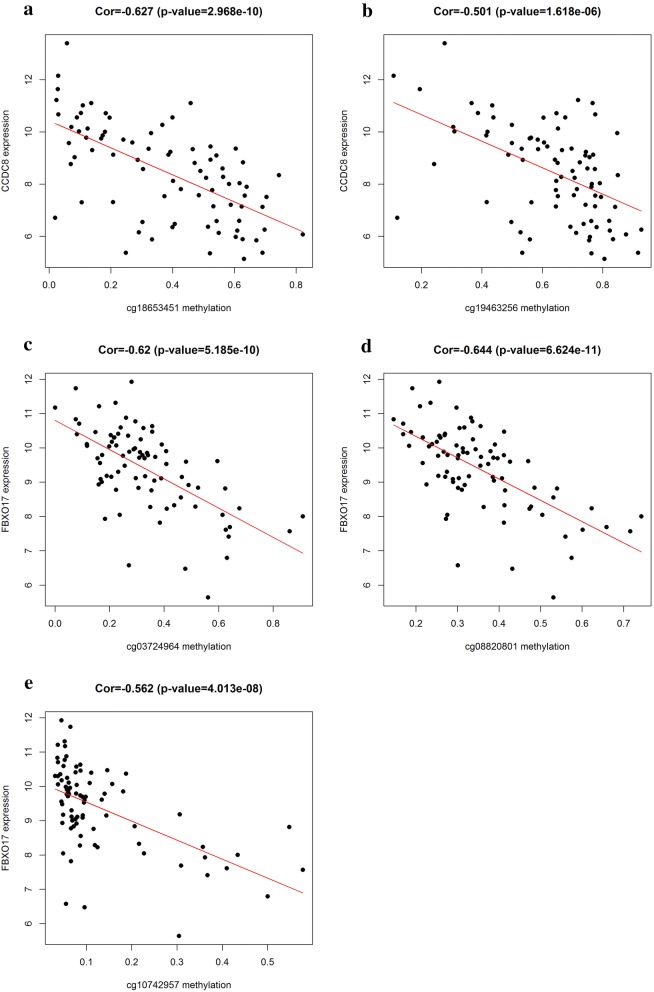
The correlation between methylated sites and gene expression. CCDC8 methylated sites with matched gene expression **a, b**. FBXO17 methylated sites with matched gene expression (**c**–**e**)

The prognostic value of 26 methylation-driven genes was assessed by Survival R package, and we found five genes (ABCD1, SLC5A10, SPIN3, ZNF69, and ZNF608) were independent prognostic indicators for ESCC (Fig. 5). However, to further investigate the correlation between genes methylation and expression, we combined these data to study the influence on patients’ survival. Using p < 0.05 as a significant standard for integrative survival, the gene expression and methylation levels of the prognostic genes ABCD1, CCDC8, FBXO17 were meaningfully correlated with prognosis (Fig. 6). Also, the prognosis-related genes methylation sites based on corresponding data in TCGA were identified, and the correlation between genes expression and sites were analyzed (Table 2). The gene expression of ABCD1, CCDC8 and FBXO17 were identified to be correlated with the methylation level of multiple sites, and all of them showed negative correlations (Figs. 7, 8).

**Table 2:** Correlation between gene expression and methylation sites

Gene symbol	Methylation site	Correlation	p value
ABCD1	cg02772106	− 0.585	7.70E−09
	cg07761358	− 0.601	2.31E−09
	cg19900558	− 0.612	9.71E−10
	cg26149887	− 0.563	3.73E−08
CCDC8	cg03576469	− 0.588	6.39E−09
	cg06747432	− 0.505	1.32E−06
	cg15984661	− 0.512	8.64E−07
	cg17375267	− 0.528	3.46E−07
	cg18653451	− 0.627	2.97E−10
	cg19463256	− 0.501	1.62E−06
FBXO17	cg03724964	− 0.62	5.19E−10
	cg08820801	− 0.644	6.62E−11
	cg10742957	− 0.562	4.01E−08

## Discussion

Esophageal carcinoma is one of the most common malignant tumors of the digestive system with high mortality and poor prognosis [18]. Esophageal adenocarcinoma (EAC) and esophageal squamous cell carcinoma (ESCC) are the major histological subtype of esophageal cancer. Alcohol consumption and tobacco smoking are two main risk factors in ESCC [19, 20], while obesity, diet and gastroesophageal reflux disease (GERD) were considered as risk indicators for EAC [21, 22]. Despite chemoradiotherapy or surgery, the prognosis of esophageal carcinoma remains poor with the overall survival [23]. The mechanism of ESCC is still unclear. Therefore, a further study of ESCC and subsequent therapeutic advances are urgently needed. Both epigenetic and genetic aberrations have been identified to ESCC generation and progression. With the rapid development of gene analysis technology, we can further study the molecular characteristics of ESCC, which provides valuable evidence for prognosis and therapeutic molecular targets.

Recently, the study on the relationship between epigenetics and tumorigenesis is always one of hotspots in the molecular biology. Epigenetics are different in that nucleotide sequences have not changed and play roles via DNA methylation, chromosome remodeling and histone deacetylation. Many studies have shown that DNA methylation is correlated with human ESCC. Aberrant DNA methylation of genes can be served as noninvasive biomarkers for the diagnosis and detection of cancer [24, 25]. Therefore, to investigate the epigenetic changes and the molecular mechanisms of ESCC progression that determines promising biomarkers, early diagnosis, treatment of ESCC is significant. The stability and independence of aberrant methylated DNA analysis make it a feasible approach for prognostic biomarkers [26]. Several reports have shown that the aberrant methylation of DNA affects genes involved in DNA damage, cell cycle, Wnt, NF-κB signaling pathways, including MGMT [27], P16 [11], DACH1 [28] and ZNF382 [29]. Also, other studies have shown that methylated FHIT is correlated with poor prognosis in early ESCC [30]. Therefore, bioinformatics analysis of the molecular functional enrichment and prognostic value of aberrant methylation DNA can offer clinicians with promising tools to predict prognosis and treat patients.

In our study, we investigated aberrant methylated genes between normal samples and ESCC patients to identify the biomarkers of prognosis related to methylation-driven genes. A model-based tool (methylmix) was used to identify those genes with abnormal methylation and correlation with gene expression, and 26 methylation-driven genes were found [13]. To study the functional roles of these ESCC methylation-driven genes, gene ontology (GO) and pathway analysis were performed. As was revealed by DAVID database, methylation-driven genes in ESCC were enriched in molecular function (MF) of DNA binding and transcription factor activity. As for cell component (CC), these genes showed enrichment in nonmotile primary cilium. Besides, biological process (BP) indicated enrichment predominantly at regulation of RNA metabolic process. These functional items not only showed the interaction of genes at the functional level but also revealed the aberration of genes function may result from abnormally methylated DNA in different samples.

In order to study further of the relationship between methylation-driven genes and patients, survival R package was utilized to analyze the correlation between abnormal DNA methylation and patients survival. Five candidate genes (ABCD1, SLC5A10, SPIN3, ZNF69, ZNF608) were identified from 26 driven genes (p < 0.05), and they might be served as independent prognostic factor for ESCC. However, it was still not comprehensive for just analyzing aberrant methylation data with patients’ survival. Thus, we moved on to combine abnormal methylation genes and the corresponding gene expression data with patients’ survival for integrative survival analysis. In the result, ABCD1, CCDC8, FBXO17 were identified to be meaningfully correlated with prognosis. Previous studies have suggested CCDC8 (coiled-coil domain containing 8) was frequently epigenetically dysregulated in renal cell carcinoma and in breast carcinomas that metastasis to the brain [31, 32]. Also, FBXO17 (F-box protein 17) have been identified to be hypermethylated in salivary gland adenoid cystic tumor [33]. For these specific genes, we further studied the correlation between expression level with methylation level of the sites, and we found multiple sites were negatively correlated with the gene expression level. The result may due to aberrant methylation of the sites leading to the dysregulation of the expression, which affects the generation and progression of cancers and the prognosis of patients.

Growing evidence demonstrated that the aberrant DNA methylation was associated tumors generation and progression via the bioinformatics analysis. For instance, Gao et al. found a prognostic risk model for evaluating the prognosis of LUSC patients, and they studied the abnormal methylated sites of key genes which had poor prognosis with patients [34]. Also, Fan et al. used GEO database to study aberrant methylation genes as biomarkers for hepatocellular cancer [35]. At present, the abnormally methylated genes in ESCC still have not been studied. Compared to previous studies, we used methylmix as a technology, which provided a more comprehensive analysis for screening methylation-driven genes in ESCC. For transferring the result to practical application, we studied the methylation driven-genes which were significant with patients’ survival. Furthermore, the correlation between abnormally methylated sites and gene expression was analyzed for providing a more precise target for further experimental validation. Although we have made comprehensive study correlated with epigenetics changes and ESCC, the experiments are still significant to testify its specificity and sensitivity.

## Conclusion

In summary, we found DNA methylation-driven genes involved in ESCC generation and progression by using methylmix technology. On this basis, we further studied the driven-genes related to patients’ survival. In the result, ABCD1, SLC5A10, SPIN3, ZNF69, ZNF608 were identified and can be served as independent prognostic factors for ESCC. ABCD1, CCDC8 and FBXO17 were screened out by the integrative survival analysis, and multiple methylated sites were correlated with gene expression. Those aberrant methylated genes may contribute to reveal the mechanisms of ESCC generation and progression and can be served as promising biomarkers for diagnosis, treatment and prognosis. Further characterization of the DNA methylated changes can help to figure out the mechanisms and design improved existing treatment.

## Additional files

**Additional file 1: Table S1.** Patients’ pathological and clinical features.

**Additional file 2: Figure S1.** The methylmix model of the rest of methylation-driven genes in ESCC.

**Additional file 3: Figure S2.** The correlation of rest of methylation-driven genes in ESCC.

## Abbreviations

TCGA: The Cancer Genome Atlas
ESCC: esophageal squamous cell carcinoma
DEG: differentially expressed gene
DMG: differentially methylated gene
GO: gene ontology
BP: biological processes
MF: molecular function
CC: cell component
GEO: gene expression omnibus
