# Supplementary material for: Identification of DNA methylation-driven genes in esophageal squamous cell carcinoma: a study based on The Cancer Genome Atlas
Source: Cancer Cell Int. 2019 Mar 6;19:52. doi: 10.1186/s12935-019-0770-9 (PMC6404309; doi:10.1186/s12935-019-0770-9)
Supplement: Supplementary file 1 — Additional file 1: Table S1. Patients’ pathological and clinical features. [file 12935_2019_770_MOESM1_ESM.docx]

| **Table S1. Patients’ pathological and clinical features.** | | | | | | | | |
| --- | --- | --- | --- | --- | --- | --- | --- | --- |
| **Id** | **Age** | **Sex** | **OS** | **T** | **N** | **M** | **Stage** | **Histology type** |
| **TCGA-LN-A49R** | 46 | MALE | 407 | T3 | N1 | M0 | Stage III | Esophagus Squamous Cell Carcinoma |
| **TCGA-Z6-A9VB** | 53 | MALE | 40 | T3 | N1 | M0 | Stage IIIA | Esophagus Squamous Cell Carcinoma |
| **TCGA-JY-A93F** | 58 | FEMALE | 731 | T2 | N0 | M0 | Stage IB | Esophagus Squamous Cell Carcinoma |
| **TCGA-LN-A8I1** | 67 | FEMALE | 401 | T2 | N0 | M0 | Stage IIA | Esophagus Squamous Cell Carcinoma |
| **TCGA-Z6-A8JE** | 57 | MALE | 64 | T3 | N1 | M0 | Stage IIIA | Esophagus Squamous Cell Carcinoma |
| **TCGA-LN-A9FQ** | 62 | MALE | 391 | T3 | N0 | M0 | Stage IIA | Esophagus Squamous Cell Carcinoma |
| **TCGA-LN-A7HY** | 50 | MALE | 366 | T3 | N1 | M0 | Stage III | Esophagus Squamous Cell Carcinoma |
| **TCGA-LN-A5U7** | 46 | MALE | 768 | T2 | N0 | M0 | Stage IIA | Esophagus Squamous Cell Carcinoma |
| **TCGA-IG-A4QT** | 56 | MALE | 283 | T3 | N0 | M0 | Stage IIA | Esophagus Squamous Cell Carcinoma |
| **TCGA-IG-A5S3** | 69 | FEMALE | 712 | T3 | N0 | M0 | Stage IIB | Esophagus Squamous Cell Carcinoma |
| **TCGA-IG-A97I** | 58 | MALE | 370 | T2 | N0 | M0 | Stage IIA | Esophagus Squamous Cell Carcinoma |
| **TCGA-LN-A49Y** | 77 | MALE | 379 | T3 | N0 | M0 | Stage IIA | Esophagus Squamous Cell Carcinoma |
| **TCGA-L5-A8NQ** | 71 | MALE | 650 | T2 | N0 | M0 | Stage IIA | Esophagus Squamous Cell Carcinoma |
| **TCGA-V5-AASV** | 67 | MALE | 467 | T3 | N0 | MX | Stage IIB | Esophagus Squamous Cell Carcinoma |
| **TCGA-VR-A8Q7** | 60 | MALE | 1590 | T3 | N1 | M0 | Stage IIIA | Esophagus Squamous Cell Carcinoma |
| **TCGA-LN-A4A2** | 57 | MALE | 380 | T3 | N0 | M0 | Stage IIA | Esophagus Squamous Cell Carcinoma |
| **TCGA-IG-A8O2** | 62 | MALE | 142 | T3 | N2 | M0 | Stage IIIB | Esophagus Squamous Cell Carcinoma |
| **TCGA-LN-A4A4** | 36 | MALE | 383 | T3 | N1 | M0 | Stage III | Esophagus Squamous Cell Carcinoma |
| **TCGA-LN-A49W** | 73 | MALE | 403 | T3 | N1 | M0 | Stage III | Esophagus Squamous Cell Carcinoma |
| **TCGA-LN-A49X** | 44 | MALE | 384 | T3 | N0 | M0 | Stage IIA | Esophagus Squamous Cell Carcinoma |
| **TCGA-IG-A50L** | 58 | MALE | 16 | T3 | N1 | M0 | Stage IIIA | Esophagus Squamous Cell Carcinoma |
| **TCGA-LN-A49U** | 62 | MALE | 467 | T3 | N0 | M0 | Stage IIA | Esophagus Squamous Cell Carcinoma |
| **TCGA-XP-A8T7** | 63 | FEMALE | 1254 | T2 | N0 | M0 | Stage IIA | Esophagus Squamous Cell Carcinoma |
| **TCGA-VR-A8EW** | 57 | MALE | 247 | T3 | N2 | M0 | Stage IIIB | Esophagus Squamous Cell Carcinoma |
| **TCGA-VR-AA7D** | 58 | MALE | 279 | T3 | NX | M0 | Stage IIIC | Esophagus Squamous Cell Carcinoma |
| **TCGA-L5-A43J** | 90 | MALE | 0 | T3 | N0 | MX | Stage IIB | Esophagus Squamous Cell Carcinoma |
| **TCGA-LN-A4A9** | 58 | MALE | 351 | T2 | N0 | M0 | Stage IIA | Esophagus Squamous Cell Carcinoma |
| **TCGA-L7-A56G** | 65 | MALE | 330 | T2 | NX | M0 | Stage IIA | Esophagus Squamous Cell Carcinoma |
| **TCGA-IG-A3I8** | 51 | FEMALE | 1012 | T3 | N0 | M0 | Stage IIA | Esophagus Squamous Cell Carcinoma |
| **TCGA-IG-A4P3** | 48 | MALE | 567 | T2 | N1 | M0 | Stage IIB | Esophagus Squamous Cell Carcinoma |
| **TCGA-S8-A6BW** | 51 | MALE | 620 | T2 | N0 | MX | Stage IB | Esophagus Squamous Cell Carcinoma |
| **TCGA-LN-A7HV** | 58 | MALE | 320 | T2 | N0 | M0 | Stage IIA | Esophagus Squamous Cell Carcinoma |
| **TCGA-Q9-A6FU** | 57 | FEMALE | 157 | T3 | N2 | M0 | Stage IIIB | Esophagus Squamous Cell Carcinoma |
| **TCGA-LN-A7HW** | 59 | MALE | 365 | T2 | N0 | M0 | Stage IIA | Esophagus Squamous Cell Carcinoma |
| **TCGA-VR-AA7I** | 70 | MALE | 484 | T4 | N0 | M0 | Stage III | Esophagus Squamous Cell Carcinoma |
| **TCGA-V5-A7RC** | 55 | MALE | 104 | T4 | NX | M1 | Stage IV | Esophagus Squamous Cell Carcinoma |
| **TCGA-XP-A8T6** | 54 | MALE | 763 | T2 | N1 | M0 | Stage IIB | Esophagus Squamous Cell Carcinoma |
| **TCGA-KH-A6WC** | 82 | MALE | 191 | T1 | N0 | M0 | Stage IA | Esophagus Squamous Cell Carcinoma |
| **TCGA-VR-A8EU** | 51 | MALE | 557 | T1 | N1 | M1 | Stage IV | Esophagus Squamous Cell Carcinoma |
| **TCGA-VR-A8EY** | 44 | FEMALE | 1025 | T3 | NX | M0 | Stage IIA | Esophagus Squamous Cell Carcinoma |
| **TCGA-VR-AA4G** | 51 | FEMALE | 549 | T2 | N2 | M0 | Stage IIIA | Esophagus Squamous Cell Carcinoma |
| **TCGA-LN-A4A8** | 52 | MALE | 472 | T2 | N0 | M0 | Stage IIA | Esophagus Squamous Cell Carcinoma |
| **TCGA-IG-A3Y9** | 72 | MALE | 26 | T4 | N0 | M0 | Stage IIIA | Esophagus Squamous Cell Carcinoma |
| **TCGA-LN-A49M** | 62 | MALE | 385 | T2 | N0 | M0 | Stage IIA | Esophagus Squamous Cell Carcinoma |
| **TCGA-LN-A4A6** | 65 | MALE | 391 | T2 | N0 | M0 | Stage II | Esophagus Squamous Cell Carcinoma |
| **TCGA-IG-A3YC** | 62 | MALE | 612 | T3 | N1 | M0 | Stage IIIA | Esophagus Squamous Cell Carcinoma |
| **TCGA-IG-A97H** | 36 | MALE | 441 | T3 | NX | M0 | Stage IIA | Esophagus Squamous Cell Carcinoma |
| **TCGA-LN-A49N** | 50 | MALE | 378 | T2 | N1 | M0 | Stage IIB | Esophagus Squamous Cell Carcinoma |
| **TCGA-L5-A8NK** | 84 | FEMALE | 412 | T3 | N0 | M0 | Stage IIA | Esophagus Squamous Cell Carcinoma |
| **TCGA-IG-A3QL** | 54 | MALE | 1071 | T2 | N0 | M0 | Stage IIA | Esophagus Squamous Cell Carcinoma |
| **TCGA-LN-A49P** | 71 | MALE | 375 | T3 | N0 | M0 | Stage IIA | Esophagus Squamous Cell Carcinoma |
| **TCGA-L5-A88W** | 67 | MALE | 764 | T3 | N0 | MX | Stage IIA | Esophagus Squamous Cell Carcinoma |
| **TCGA-IG-A3YB** | 61 | MALE | 80 | T3 | N1 | M0 | Stage IIIA | Esophagus Squamous Cell Carcinoma |
| **TCGA-LN-A7HZ** | 49 | MALE | 401 | T2 | N0 | M0 | Stage IIA | Esophagus Squamous Cell Carcinoma |
| **TCGA-IG-A3YA** | 53 | MALE | 632 | T4 | N0 | M0 | Stage IIIA | Esophagus Squamous Cell Carcinoma |
| **TCGA-LN-A8HZ** | 56 | MALE | 375 | T2 | N0 | M0 | Stage IIA | Esophagus Squamous Cell Carcinoma |
| **TCGA-IG-A5B8** | 72 | MALE | 24 | T3 | N0 | M0 | Stage IB | Esophagus Squamous Cell Carcinoma |
| **TCGA-Z6-A8JD** | 53 | MALE | 104 | T3 | N0 | M0 | Stage IIB | Esophagus Squamous Cell Carcinoma |
| **TCGA-L5-A43H** | 75 | MALE | 9 | T3 | N1 | M0 | Stage III | Esophagus Squamous Cell Carcinoma |
| **TCGA-Z6-AAPN** | 57 | MALE | 81 | T3 | N0 | M0 | Stage IIA | Esophagus Squamous Cell Carcinoma |
| **TCGA-VR-A8EX** | 63 | MALE | 855 | T1 | N1 | M1a | Stage IVA | Esophagus Squamous Cell Carcinoma |
| **TCGA-LN-A49K** | 66 | MALE | 180 | T3 | N0 | M0 | Stage IIA | Esophagus Squamous Cell Carcinoma |
| **TCGA-LN-A9FO** | 42 | MALE | 4 | T2 | N0 | M0 | Stage IIA | Esophagus Squamous Cell Carcinoma |
| **TCGA-JY-A6FA** | 51 | MALE | 1361 | T2 | N1 | M0 | Stage IIB | Esophagus Squamous Cell Carcinoma |
| **TCGA-LN-A49L** | 44 | MALE | 153 | T2 | N0 | M0 | Stage IIA | Esophagus Squamous Cell Carcinoma |
| **TCGA-L5-A88Z** | 70 | FEMALE | 225 | T1 | N1 | M0 | Stage IIA | Esophagus Squamous Cell Carcinoma |
| **TCGA-LN-A4A1** | 60 | MALE | 383 | T3 | N0 | M0 | Stage IIA | Esophagus Squamous Cell Carcinoma |
| **TCGA-L5-A88S** | 84 | MALE | 471 | T3 | N0 | MX | Stage IB | Esophagus Squamous Cell Carcinoma |
| **TCGA-LN-A49O** | 47 | MALE | 408 | T3 | N0 | M0 | Stage IIA | Esophagus Squamous Cell Carcinoma |
| **TCGA-LN-A49V** | 49 | MALE | 383 | T3 | N0 | M0 | Stage IIA | Esophagus Squamous Cell Carcinoma |
| **TCGA-JY-A6FG** | 50 | MALE | 1263 | T3 | N1 | M0 | Stage III | Esophagus Squamous Cell Carcinoma |
| **TCGA-L5-A4OM** | 54 | FEMALE | 1458 | T1 | N0 |  | Stage IA | Esophagus Squamous Cell Carcinoma |
| **TCGA-LN-A4A5** | 49 | MALE | 681 | T2 | N0 | M0 | Stage IIA | Esophagus Squamous Cell Carcinoma |
| **TCGA-XP-A8T8** | 49 | MALE | 437 | T1 | N1 | M0 | Stage IIB | Esophagus Squamous Cell Carcinoma |
| **TCGA-LN-A9FR** | 70 | MALE | 373 | T2 | N1 | M0 | Stage IIB | Esophagus Squamous Cell Carcinoma |
| **TCGA-LN-A7HX** | 72 | MALE | 372 | T2 | N0 | M0 | Stage IIA | Esophagus Squamous Cell Carcinoma |
| **TCGA-LN-A8I0** | 52 | MALE | 407 | T2 | N0 | M0 | Stage IIA | Esophagus Squamous Cell Carcinoma |
| **TCGA-VR-A8EO** | 49 | MALE | 785 | T3 | N0 | M0 | Stage IIA | Esophagus Squamous Cell Carcinoma |
| **TCGA-IG-A51D** | 63 | MALE | 518 | T2 | NX | M1 | Stage IIB | Esophagus Squamous Cell Carcinoma |
| **TCGA-JY-A6FE** | 49 | MALE | 112 | T3 | N1 | M0 | Stage III | Esophagus Squamous Cell Carcinoma |
| **TCGA-LN-A9FP** | 60 | FEMALE | 366 | T2 | N0 | M0 | Stage IIA | Esophagus Squamous Cell Carcinoma |
| **TCGA-IG-A6QS** | 54 | MALE | 303 | T2 | N1 | M0 | Stage IIB | Esophagus Squamous Cell Carcinoma |
| **TCGA-LN-A4A3** | 61 | MALE | 388 | T3 | N1 | M0 | Stage III | Esophagus Squamous Cell Carcinoma |
| **TCGA-LN-A4MQ** | 46 | MALE | 375 | T3 | N1 | M0 | Stage III | Esophagus Squamous Cell Carcinoma |
| **TCGA-VR-A8ET** | 64 | MALE | 47 | T2 | NX | M0 | Stage IIA | Esophagus Squamous Cell Carcinoma |
| **TCGA-LN-A5U5** | 57 | MALE | 136 | T3 | N1 | M1 | Stage IV | Esophagus Squamous Cell Carcinoma |
| **TCGA-LN-A49S** | 59 | MALE | 400 | T3 | N0 | M0 | Stage IIA | Esophagus Squamous Cell Carcinoma |
| **TCGA-VR-AA7B** | 65 | FEMALE | 342 | T3 | NX | M0 | Stage IV | Esophagus Squamous Cell Carcinoma |
| **TCGA-VR-A8ER** | 54 | MALE | 378 | T4 | N1 | M0 | Stage III | Esophagus Squamous Cell Carcinoma |
| **TCGA-VR-A8EZ** | 47 | MALE | 553 | T3 | NX | M0 | Stage IIIC | Esophagus Squamous Cell Carcinoma |
| **TCGA-VR-A8EP** | 51 | MALE | 824 | T3 | NX | M0 | Stage IIIB | Esophagus Squamous Cell Carcinoma |
| **TCGA-JY-A6FD** | 51 | FEMALE | 2069 | T3 | N0 | M0 | Stage IIA | Esophagus Squamous Cell Carcinoma |
| **TCGA-LN-A5U6** | 54 | MALE | 375 | T2 | N1 | M0 | Stage IIB | Esophagus Squamous Cell Carcinoma |
| **TCGA-IC-A6RF** | 69 | FEMALE | 477 | T1 | N1 | M0 | Stage IA | Esophagus Squamous Cell Carcinoma |
| **TCGA-LN-A4MR** | 57 | MALE | 402 | T2 | N0 | M0 | Stage IIA | Esophagus Squamous Cell Carcinoma |
| **TCGA-IG-A625** | 60 | MALE | 390 | T3 | N2 | M0 | Stage IIIB | Esophagus Squamous Cell Carcinoma |
