# Supplementary figures and images for: Identification of DNA methylation-driven genes in esophageal squamous cell carcinoma: a study based on The Cancer Genome Atlas
Source: Cancer Cell Int. 2019 Mar 6;19:52. doi: 10.1186/s12935-019-0770-9 (PMC6404309; doi:10.1186/s12935-019-0770-9)

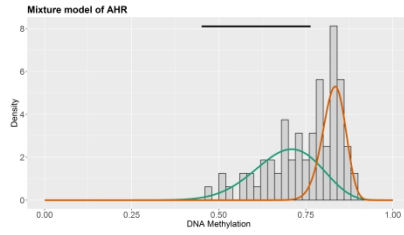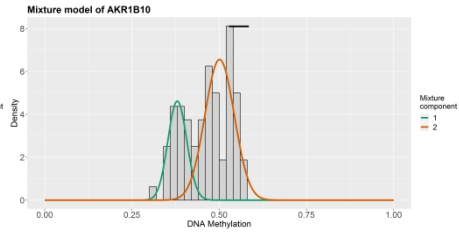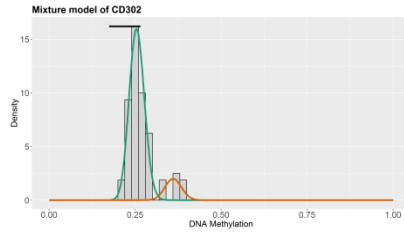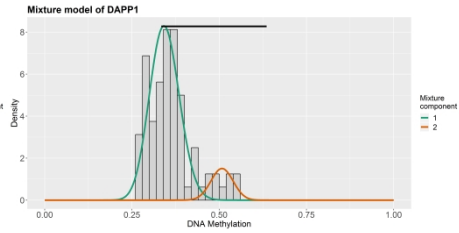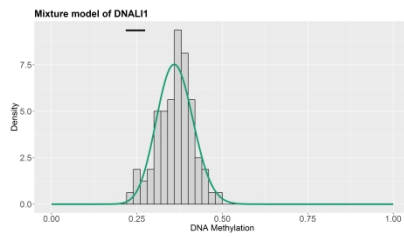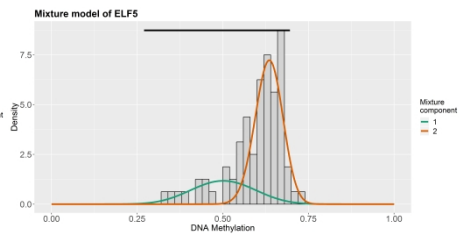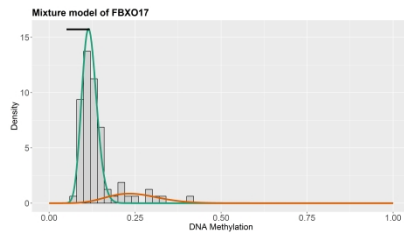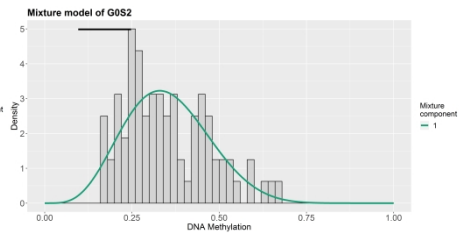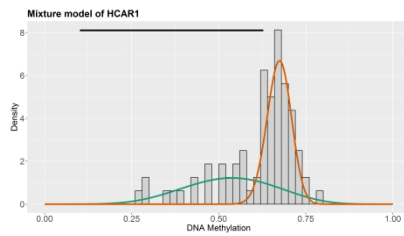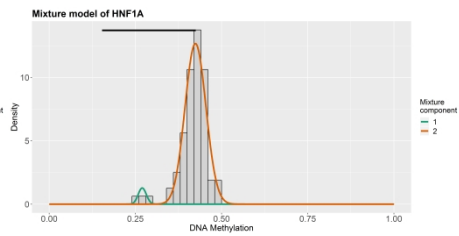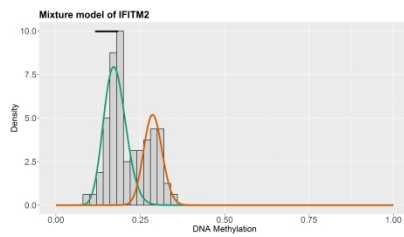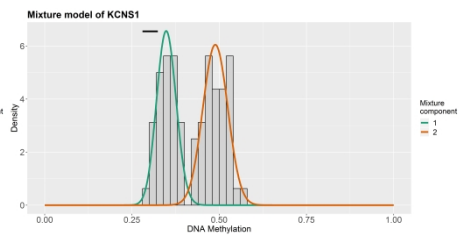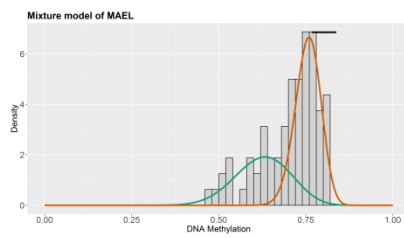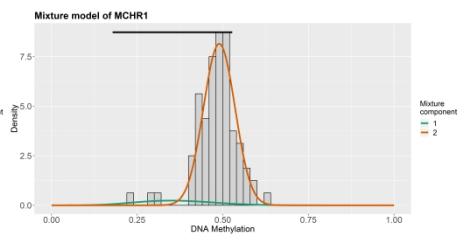

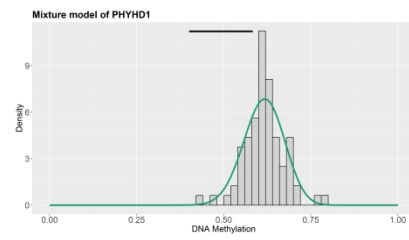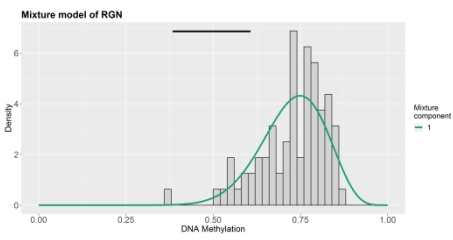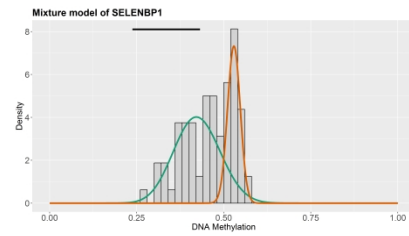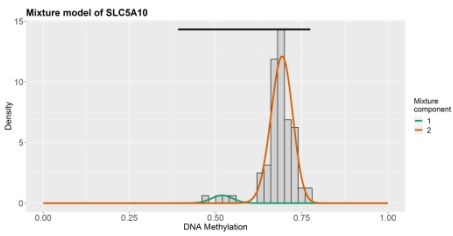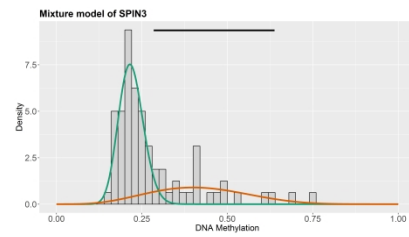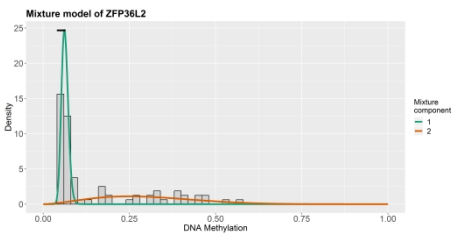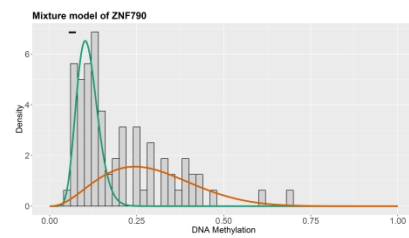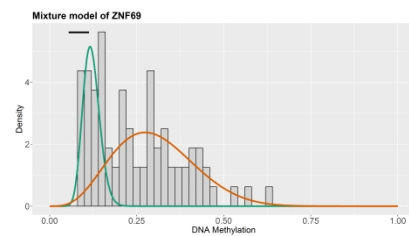

Supplement: Supplementary file 2 — Additional file 2: Figure S1. The methylmix model of the rest of methylation-driven genes in ESCC. [file 12935_2019_770_MOESM2_ESM.pdf]

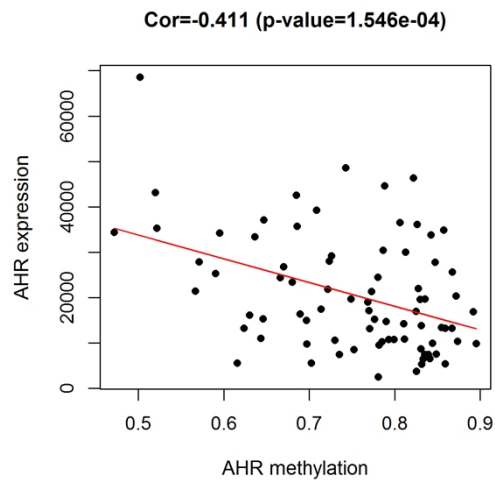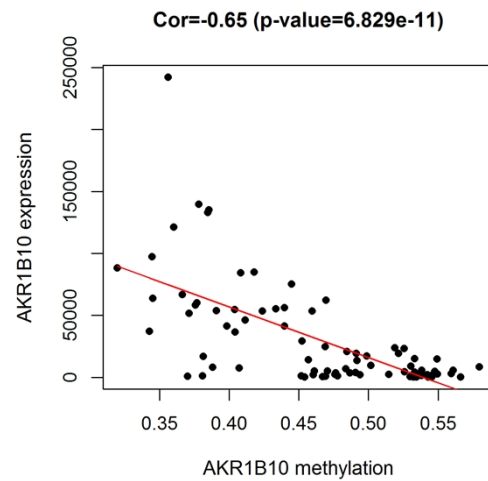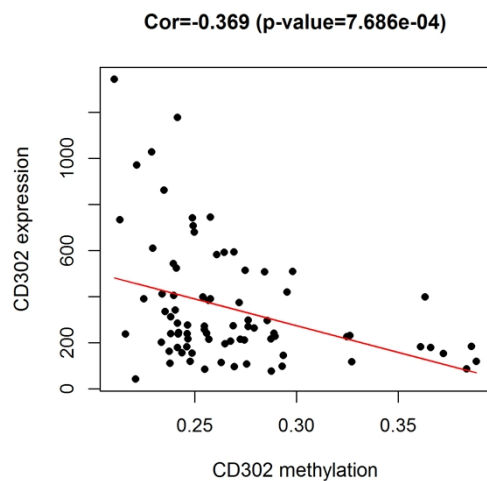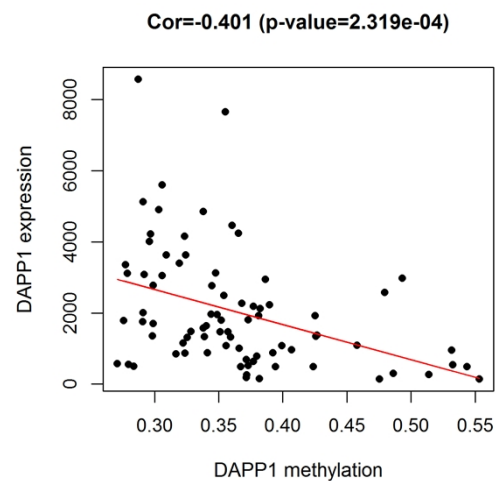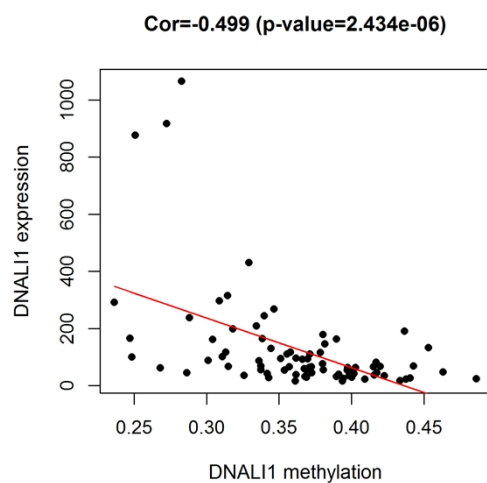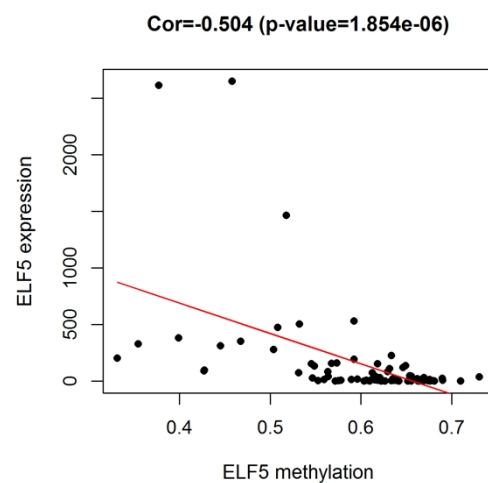

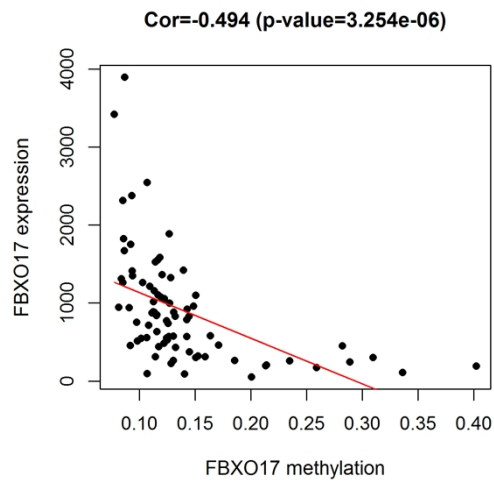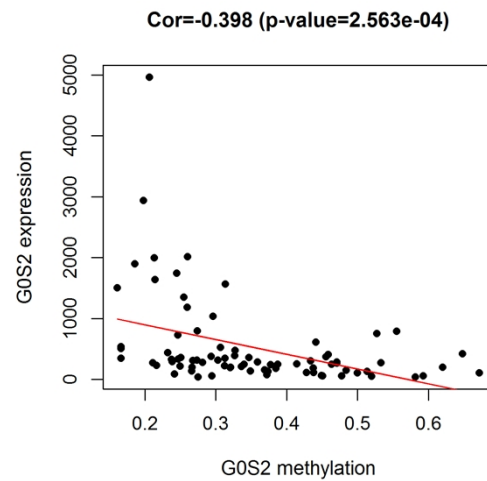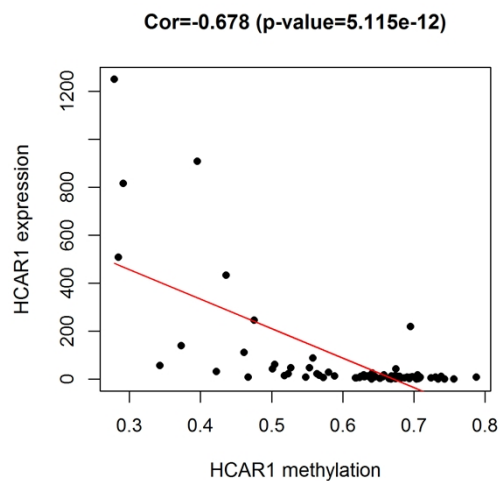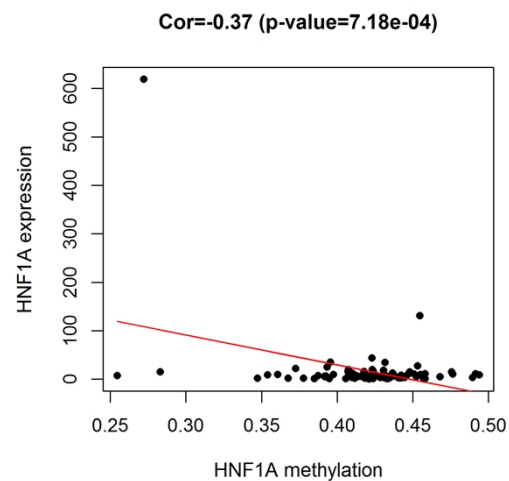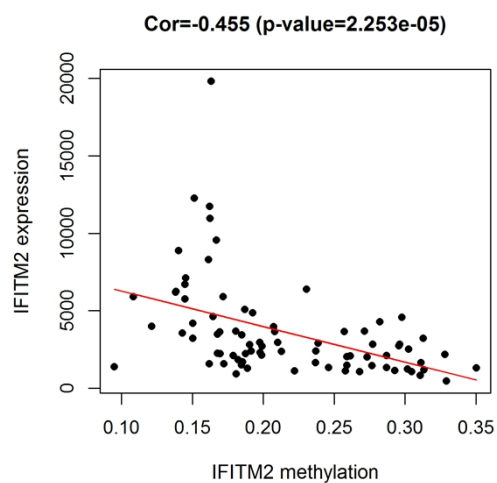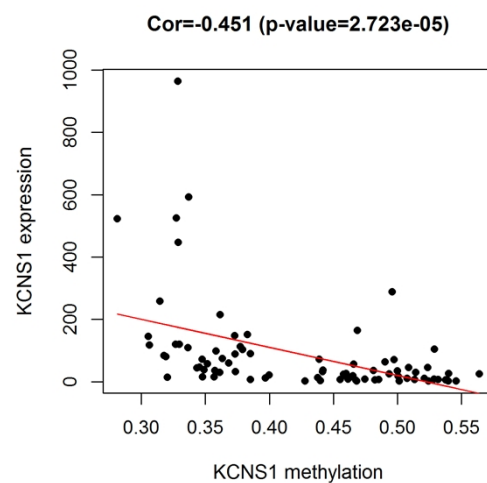

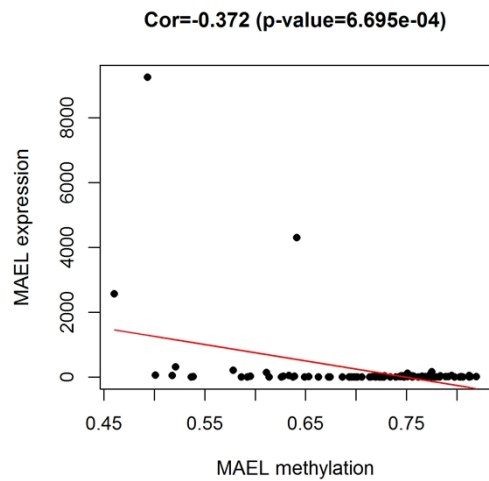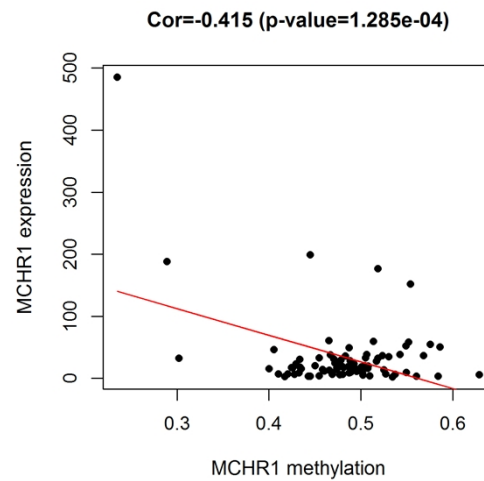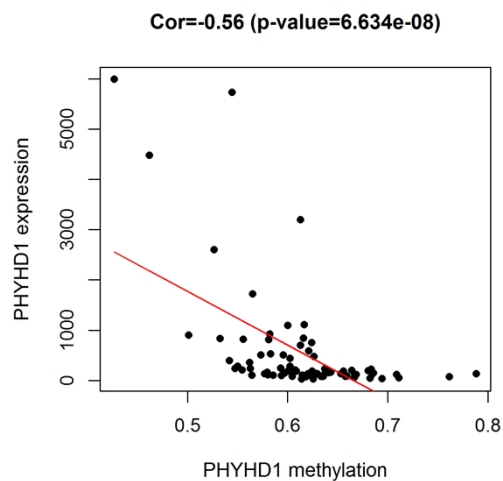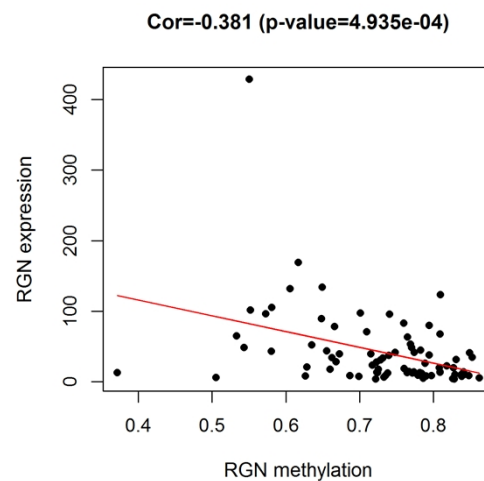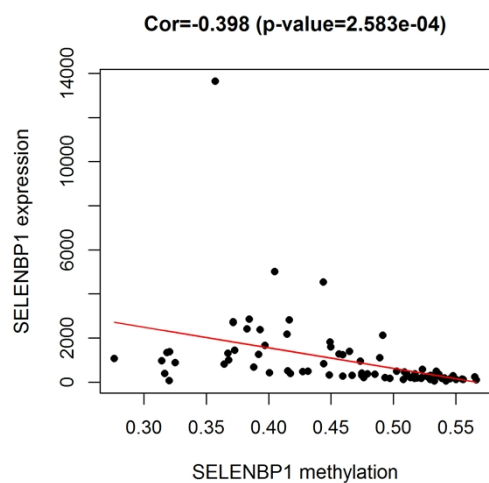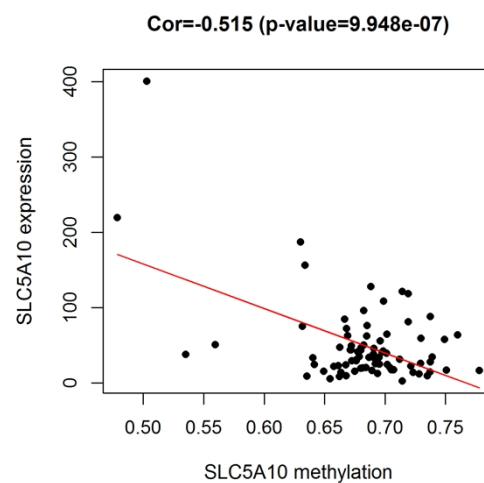

Cor=-0.381 (p-value=4.877e-04)

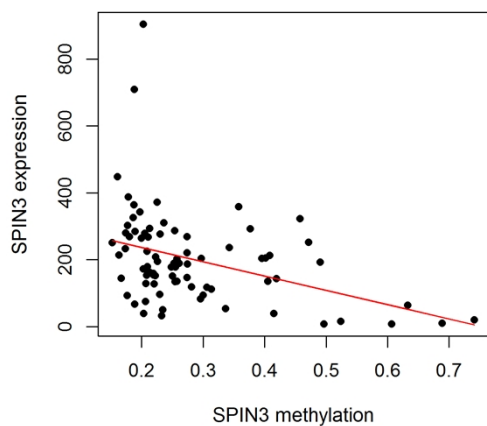

Cor=-0.37 (p-value=7.282e-04)

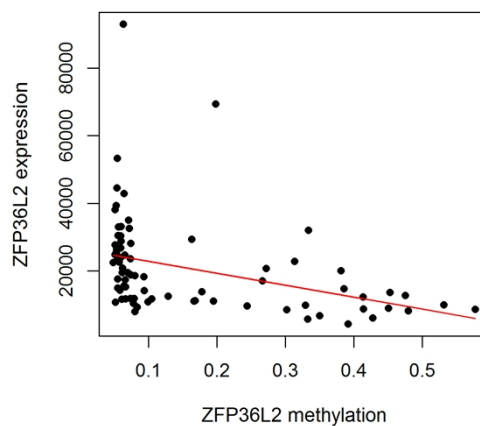

Cor=-0.505 (p-value=1.82e-06)

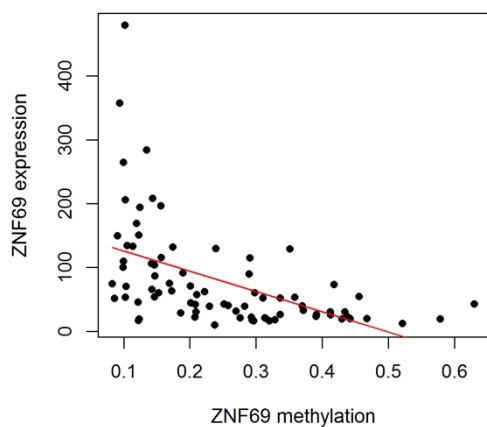

Cor=-0.401 (p-value=2.313e-04)

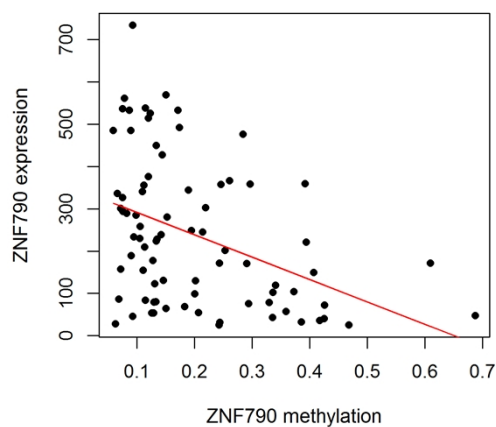

Supplement: Supplementary file 3 — Additional file 3: Figure S2. The correlation of rest of methylation-driven genes in ESCC. [file 12935_2019_770_MOESM3_ESM.pdf]
